# Changes and sex differences in patient reported outcomes in rheumatoid factor positive RA–results from a community based study

**DOI:** 10.1186/1471-2474-15-44

**Published:** 2014-02-20

**Authors:** Korosh Hekmat, Lennart T Jacobsson, Jan-Åke Nilsson, Ylva Lindroth, Carl Turesson

**Affiliations:** 1 Department of Clinical Sciences, Section of Rheumatology, Lund University, Malmö, Sweden

## Abstract

**Background:**

Patient reported outcomes (PROs) are important measures in rheumatoid arthritis (RA). A register of patients with RA from all rheumatology care providers in Malmö, Sweden, was established in 1997 and has been continually updated. This register includes virtually all the RA patients in the area. The aim of this study was to analyse PROs in surveys of this population conducted between 1997 and 2009, and to assess differences in treatment and outcome in male and female patients.

**Method:**

In 1997, 2002, 2005 and 2009, questionnaires were sent to the RA patients in the register (n = 1016 in 1997; n = 916 in 2002; n = 1625 in 2005; n = 1700 in 2009). Response rates varied between 62 % and 74 %, and 72-74 % was women. Questionnaire data included medication and measures of disability and health related quality of life. Data on rheumatoid factor (RF) tests were retrieved from the databases of the two clinical immunology laboratories in the area. In order to limit the impact of changes in the case mix over time, the study was restricted to RF positive patients. The analyses were stratified by sex.

**Results:**

Patients reported less severe outcomes for all measures in the later surveys compared to 1997, and patients’ global disease activity assessment and self-reported pain were further improved in 2009 compared to 2005. Treatment with biologics increased over time from 1997 (none) to 2009 (29%), with no difference between men and women. Visual analogue scales (0-100) for patients’ global assessment of disease activity [mean 45 (95 % CI (45-47) vs. 38 (35-40)] and pain [mean 46 (44-49) vs. 38 (36-40)] decreased from 1997 to 2009, with numerically greater improvement in male patients. The mean SF-36 physical component scores also improved, and were higher in men than in women in all surveys.

**Conclusion:**

Pharmacologic treatment of RA became more extensive over time, and there was improvement in all PROs. Despite similar treatment, male patients reported better outcomes, in particular for pain and physical function, compared to female patients. We suggest that patient reported outcomes should be reported separately in male and female patients with RA.

## Background

Rheumatoid arthritis (RA) is a chronic, inflammatory disorder, which is characterized by progressive joint damage and has a major impact on physical function and health related quality of life (HRQoL). There is evidence indicating that the disease has become less severe during the last decades, possibly due to better management with more extensive treatment, or secular changes in other factors influencing disease severity. For example, a lower disease activity and disability in 1995 compared to 1978 has been demonstrated in Swedish patients with RA [1], and a recent survey indicated that the incidence of total hip arthroplasties has decreased in RA patients over time [2]. Furthermore, severe extra-articular RA manifestations such as vasculitis have also become less frequent in recent years [3]. On the other hand, in poor countries many patients still have active, uncontrolled disease [4].

A patient-reported outcome (PRO) is a questionnaire used in a clinical trial or a clinical setting, where the responses are collected directly from the patient. A number of PROs are validated measures that are considered relevant outcomes in quantitative research [5] and feasible, quantitative measures for standard rheumatology clinical care [6]. RA is far more common in women, and sex specific factors may also influence various aspects of disease severity, including PROs. For example, there seem to be gender differences with worse reported HRQoL among female patients with early RA [7], and a fourfold increased risk of work disability in women with RA compared to men [8]. The impact of recent changes in management and recent secular changes on such differences is unknown.

Our aim was to investigate changes over time in PROs such as visual analogue scales (VAS) for patients’ global assessment of disease activity and pain, disability and HRQoL, as well as treatment in male and female patients in a population-based sample of patients with RA.

## Methods

### Patients

In 1997, a register of all known patients with RA in the city of Malmö, Sweden, was established. Inclusion was based on a clinical diagnosis of RA by a rheumatologist and fulfilment of the 1987 American College of Rheumatology (ACR) criteria for RA [9].

The corresponding background population of Malmö was 251,000 in 1997. Patients were recruited from the rheumatology outpatient clinic of Malmö University Hospital (as of 2010 a part of Skåne University Hospital), which is the only hospital serving the city, and from the four rheumatologists in private practice in Malmö [10]. The close collaboration between the university clinic and the private practitioners and the methods for recruiting patients to the register, which has been continuously updated after 2002, have been described in detail previously [10,11,2].

The prevalence of RA in the area (approximately 0.5 % among those aged 20 years and above) and the sex and age distributions in the Malmö RA register were found to be comparable to the RA prevalence in a study from a population-based RA register in Oslo, Norway [12]. Subsequent surveys using the diagnostic index of primary care centres and questionnaires sent to other physicians in the area indicate that >90% of all patients with diagnosed RA in the city at that time were included in the register. All registered cases with RA were validated by review of the case records as previously described [11,2].

### Variables

In 1997, 2002, 2005 and 2009, self-administered questionnaires were sent to the patients in the Malmö RA register. Demographics, working status, medication with disease modifying anti-rheumatic drugs (DMARDs), visual analogue scales (VAS) for general health and pain, use of healthcare, the Swedish version of the health assessment questionnaire (HAQ) [13], and HRQoL as measured by the Swedish version of the short form (SF)-36 were assessed [14,15].

SF-36 is a generic measure of eight health dimensions (physical functioning, physical role, bodily pain, general health, vitality, social functioning, emotional role, mental health) with scale from 0 to 100 (0 worst health). A reminder was sent to patients who did not answer the questionnaire the first time.

The study was approved by the regional research ethics committee in Lund, Sweden. Data on rheumatoid factor (RF) tests were retrieved from the databases of the two clinical immunology laboratories in the area. In order to limit the impact of changes in the case mix, in particular regarding mild, RF negative cases, over time, in the present comparison, only patients with at least one positive RF test were included. The analyses were stratified by sex.

### Statistical analysis

Data were analyzed with version 18.0 of the Statistical Package for the Social Sciences (SPSS Inc., Chicago, IL). As the questionnaire data in 1997, 2002, 2005 and 2009 included partly overlapping patient populations, no formal statistical comparisons were made between these patient cohorts. Too few had responded to repeated surveys to allow meaningful longitudinal data analyses. Variables with a normal distribution are presented as means with 95% confidence intervals (95% CI) whereas those with a non-normal distribution are presented as medians with interquartile ranges (IQR). As a conservative estimate we assumed that a significant change had occurred if the 95% CIs of a measure in 1997 and at subsequent years of examination did not overlap. The SF-36 scores were compared to the expected derived from normative values from the Swedish population [16] for each individual, and mean differences from the expected with 95 % CI were calculated for each survey. CIs not including zero were interpreted as indicating a significant difference from the expected.

## Results

In 1997, 2002, 2005 and 2008, questionnaires were sent to the RA patients in the register (n = 1016 in 1997; n = 916 in 2002; n = 1625 in 2005 and n = 1700 in 2009). Overall response rates were 74 %, 66 %, 64 % and 62 %, respectively. The demographics of responders with at least one positive test for RF are shown in Table 1. There was no major difference in disease duration and age between the surveys (Table 1).

**Table 1:** Demographics, treatment and patient reported outcomes in four surveys of RF positive patients in the Malmö RA population

	**1997**	**2002**	**2005**	**2009**
N	668	438	517	454
Disease duration years, mean (SD)	15.0 (13.6)	16.7 (12.5)	15.8 (12.5)	17.2 (12.1)
Female sex	497 (74%)	321 (73%)	368 (71%)	331 (73%)
Age; years, mean	61.9 (14.1)	63.9 (13.6)	62.9 (14.2)	63.8 (13.4)
**Current treatment** proportion (95 % CI)				
Corticosteroids	19% (16-22)	30% (26-35)	26 % (23-30)	31 % (27-35)
Biologic	0	16% (12-19)	23 % (19-27)	29 % (25-33)
Methotrexate	20% (17-23)	44% (40-49)	56 % (52-60)	58 % (54-63)
**Patient reported outcomes** mean (95% CI) unless otherwise noted				
HAQ*	1.12 (0.50-1.75)	1.00 (0.50-1.62)	0.88 (0.38-1.38)	0.88 (0.38-1.5)
VAS global	44.8 (42.3-47.4)	40.0 (37.6-42.4)	41.8 (39.6-44.1)	37.6 (35.2-40.0)
VAS pain	46.3 (43.7-48.9)	41.1 (38.8-43.5)	40.8 (38.6-43.0)	38.2 (35.8-40.7)
SF-36 PCS	32.1 (31.0-33.1)	33.2 (32.2-34.4)	34.6 (33.6-35.7)	35.2 (34.0-36.4)
SF-36 MCS	45.4 (44.0-46.7)	46.7 (45.4-48.1)	47.9 (46.7-49.0)	47.1 (45.9-48.3)

* median (IQR).

PCS = Physical component score.

MCS = Mental component score.

### Temporal trends in treatment and PROs

**Figure 1:**
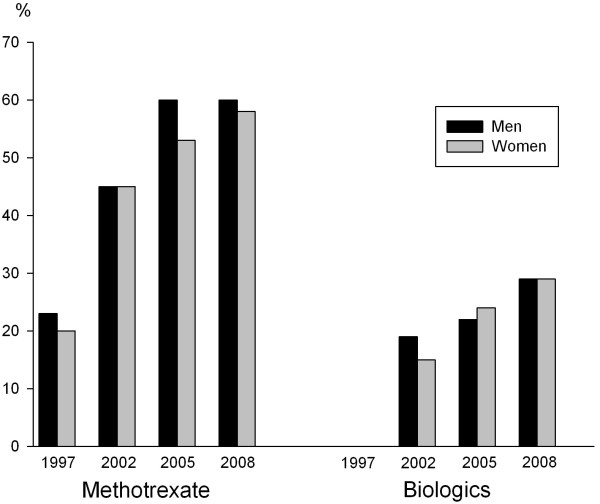
Current treatment with methotrexate and biologics in RF positive rheumatoid arthritis patients in 1997, 2002. 2005 and 2009, by sex.

More patients were treated with methotrexate in 2005 and 2009 compared to 1997 (Figure 1).

As expected, treatment with TNF inhibitors and other biologics was only reported in 2002 and later. Reported current treatment with biologics increased gradually over time from 2002 to 2009, without major differences between male and female patients (Figure 1). In 2009, 29% of patients of both sexes were treated with biologics. Triple therapy with methotrexate, sulphasalazine and antimalarials was used in <1% in 1997, 2002 and 2009. For 2005 data on triple therapy were not available.

**Figure 2:**
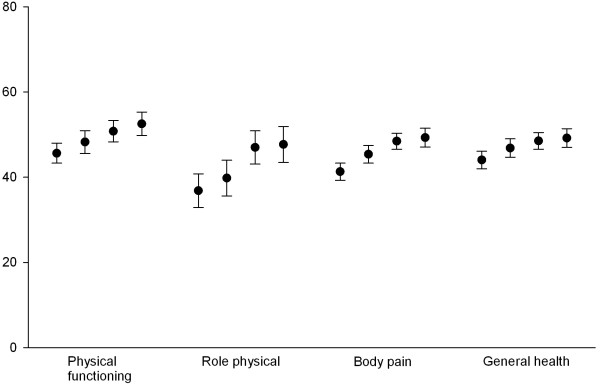
**SF-36 Physical health related scales in RF positive rheumatoid arthritis patients in 1997, 2002. 2005 and 2009.** Means with 95% confidence intervals.

**Figure 3:**
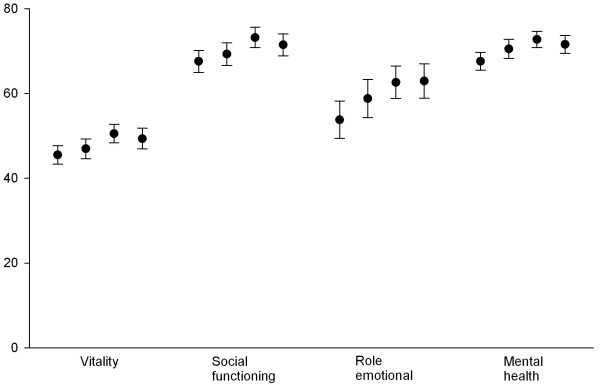
**SF-36 Mental health related scales in RF positive rheumatoid arthritis patients in 1997, 2002. 2005 and 2009.** Means with 95% confidence intervals.

Patients’ global assessment of disease activity and pain both decreased substantially from 1997 to 2009 (Table 1). There was a similar trend for HAQ, with the exception of 2005 and 2009, when the median HAQ score was stable (Table 1). The mean SF-36 physical component scores were substantially better in the later surveys (Table 1). In particular, there was improvement over time in the physical health related scales for physical functioning, role physical and body pain (Figure 2). There were similar, although more modest, changes in the SF-36 mental component score (Table 1) and the mental health related scores (Figure 3). Despite these improvements, even in 2009 the scores were significantly lower compared to the expected derived from normative values from the Swedish population (mean difference with 95% CI for Physical functioning 21.9 (19.3-24.4), Role physical 21.2 (17.0-25.5) Body pain 18.4 (16.3-20.5), General health 19.0 (16.8-21.3), Vitality 15.6 (13.1-18.1), Social functioning 13.6 (11.0-16.2), Role-emotion 15.2 (11.1-19.2) and Mental health 7.4 (5.3-9.5)).

### Sex differences in PROs

There was no major difference in age between male and female responders in either of the surveys (Table 2). Male patients reported better outcomes with lower point estimates for VAS global, VAS pain and HAQ and higher point estimates for SF-36 PCS and SF-36 MCS (Table 2) in all four surveys (Table 2). In addition, the numerical differences for all these outcomes between mean values in 2009 vs 1997 were greater for men than women, especially for VAS pain (difference 11.5 in men vs. 7.0 in women) and VAS global (difference 9.2 in men vs. 7.1 in women) (Table 2). When examining SF-36 domain scales individually, a similar pattern was seen for all domains, with higher values for male patients than for females at all time points (Table 3). The difference between men and women in physical functioning was consistent in all surveys, with non-overlapping confidence intervals, and increased numerically over time (Table 3). For role physical, bodily pain and vitality, similar increasing differences were seen over time, with consistently higher values in men, in particular in the latter two surveys (where the CI were non-overlapping compared to women) (Table 3). The difference in mental component scores was smaller with less change over time (Table 2). In all four surveys, female as well as male patients with RA had lower scores for all SF-36 domains compared to the expected derived from sex-specific population based normative values, with CIs not overlapping zero for all differences, and numerically greater differences for women (data not shown).

**Table 2:** Demographics and patient reported outcomes in four surveys of RF positive patients in the Malmö RA population, by sex

	**1997**		**2002**		**2005**		**2009**	
	**WOMEN**	**MEN**	**WOMEN**	**MEN**	**WOMEN**	**MEN**	**WOMEN**	**MEN**
**N**	497	171	321	117	362	148	326	120
**Age**	62.0	61.8	64.0	63.6	62.8	63.2	63.8	63.7
Mean (SD)	(14.2)	(13.7)	(14.0)	(12.6)	(14.8)	(12.9)	(13.9)	(12.3)
**HAQ**	1.4	1.0	1.1	0.6	1.0	0.5	1.0	0.4
Median (IQR)	(0.6-2.0)	(0.1-1.3)	(0.6-1.8)	(0.1-1.4)	(0.5-1.6)	(0.0-1.0)	(0.5-1.6)	(0.0-1.1)
**VAS pain**	48.3	40.4	43.2	35.7	43.2	34.4	41.3	28.9
	(45.3-51.4)	(35.3-45.6)	(40.4-45.9)	(31.3-40.0)	(40.6-45.8)	(30.2-38.5)	(38.4-44.1)	(24.6-33.1)
**VAS global**	46.4	40.5	41.6	35.6	43.8	36.6	39.3	31.3
	(43.4-49.3)	(35.5-45.5)	(38.8-44.5)	(31.3-39.8)	(41.2-46.5)	(32.3-40.8)	(36.5-42.2)	(27.0-35.5)
**SF-36 PCS**	30.8	35.6	32.3	35.8	33.2	38.3	33.8	39.7
	(29.7-32.0)	(33.5-37.7)	(31.1-33.6)	(33.7-38.0)	(32.0-34.4)	(36.3-40.3)	(32.4-35.1)	(37.5-41.9)
**SF-36 MCS**	44.8	46.9	46.5	47.5	47.1	50.0	46.4	49.2
	(43.2-46.4)	(44.4-49.4)	(44.8-48.1)	(45.1-49.9)	(45.7-48.5)	(47.9-57.1)	(45.0-47.9)	(46.9-51.5)

All values are means (95% CI) unless otherwise noted.

PCS = Physical component score.

MCS = Mental component score.

**Table 3:** SF-36 domain scores in four surveys of RF positive patients in the Malmö RA population, by sex

	**1997**		**2002**		**2005**		**2009**	
**SF-36 domain**	**WOMEN**	**MEN**	**WOMEN**	**MEN**	**WOMEN**	**MEN**	**WOMEN**	**MEN**
**Physical functioning**	43.1	52.8	46.2	53.8	47.0	60.7	49.9	61.9
	(40.4-45.8)	(48.2-57.4)	(43.2-49.2)	(48.6-59.0)	(44.2-49.9)	(56.1-65.3)	(46.8-52.9)	(56.6-67.2)
**Role physical**	33.6	45.7	36.7	47.7	42.6	58.5	43.5	62.2
	(29.1-38.1)	(37.6-53.8)	(31.8-41.6)	(38.7-55.7)	(38.1-47.2)	(51.1-65.8)	(38.6-48.3)	(54.4-70.1)
**Body pain**	39.6	46.1	44.1	49.0	46.4	53.9	46.2	58.3
	(37.4-41.9)	(41.9-50.3)	(41.7-46.5)	(45.2-52.9)	(44.2-48.6)	(50.4-57.5)	(43.8-48.6)	(53.8-62.7)
**General health**	42.7	48.0	45.4	50.6	46.8	53.1	47.9	54.0
	(40.4-45.0)	(44.0-52.0)	(42.9-47.9)	(46.4-54.8)	(44.5-49.1)	(49.3-56.9)	(45.4-50.5)	(49.7-58.3)
**Vitality**	44.5	48.5	45.7	50.4	48.2	56.2	47.2	56.8
	(41.9-47.0)	(44.1-53.0)	(43.0-48.4)	(45.7-55.2)	(45.7-50.7)	(52.2-60.3)	(44.4-49.9)	(51.7-61.9)
**Social functioning**	66.1	71.9	68.9	70.4	70.6	80.2	69.9	77.4
	(63.1-69.2)	(67.0-76.9)	(65.8-72.1)	(65.2-75.6)	(67.6-73.5)	(76.4-84.1)	(66.8-73.0)	(72.5-82.3)
**Role-emotional**	51.0	61.6	57.7	61.9	59.8	70.3	59.9	72.5
	(45.9-56.1)	(53.1-70.1)	(52.4-63.0)	(53.7-70.1)	(55.1-64.5)	(63.3-77.3)	(55.0-64.7)	(65.0-79.9)
**Mental health**	66.8	76.0	69.7	77.6	70.9	72.9	70.1	69.9
	(64.3-62.3)	(71.9-80.0)	(67.0-72.3)	(74.1-81.0)	(68.6-73.3)	(68.8-77.0)	(67.7-72.6)	(65.8-74.0)

All values are means (95% CI).

## Discussion

In this study, we found that treatment with biologics and corticosteroids increased over time, and there was improvement in all patient reported outcomes. Despite similar treatment, male patients reported better outcomes and more improvement, in particular for the impact of pain, compared to female patients.

Several previous studies have reported improved outcomes over time in comparisons of samples of patients with RA evaluated at different time points. Such improvements were observed in patients’ clinical status according to disease activity [1,17], functional capacity [17-19], radiographic scores [17,20], and other clinical measures [17]. This most likely at least partly reflects the fact that management and treatment of RA has become more efficient in recent years. Several clinical trials have shown that tight control of disease activity can be achieved in many patients with early RA by optimizing treatment with traditional DMARDs [21,22]. Current guidelines emphasize the importance of a treat to-target approach [23], with addition of TNF-inhibitors and other biologics in refractory cases or as first line therapy in patients with a severe prognosis [24,25]. Currently, decision making about treatment of RA in the studied population and in other parts of Sweden is largely based on national guidelines [25]. In Malmö, initiation of treatment with biologics is discussed at a scheduled meeting, to ensure uniform indications in clinical practice.

Triple therapy with methotrexate, sulphasalazine and antimalarials was rarely used in this cohort. The low use of triple therapy in 2009 was similar to that observed in 2010 in a cross sectional study from Kristiansand, Norway [26], but lower than that reported from Jyväskylä, Finland, where biologics were less extensively used [26]. The comparison between the cohorts from Kristiansand and Jyväskylä suggested that good functional status may be reached with combination of conventional DMARDs [26].

Improvement of PROs in patients with RA is of major clinical relevance. Not only are reduced disability and improved HRQoL important treatment goals in themselves, but such measures also predict long term outcomes. For example, disability, measured using HAQ, has been shown to predict mortality in several studies [4,27,28].

In the survey performed in 2009, patients with RA had significantly lower scores for physical as well as mental components of HRQoL compared to the expected based on normative data. This suggests that although patients with RA surveyed in 2009 were significantly improved compared to previous investigations in 1997, 2002 and 2005, there is still a major difference in HRQoL compared to the general population. Evidently, there is still need for further improvements in the management of RA.

Influence of gender on various aspects of disease severity has been described in several studies. One retrospective review of a community based sample revealed a higher proportion with erosive disease in male patients, but a greater number of orthopaedic procedures in women [29]. In studies of the Swedish multi-centre early RA BARFOT cohort, women had slightly higher disease activity, measured using the DAS28 score, compared to men already at baseline [30,31], mainly due to higher numbers of tender joints and worse rating of general health [31]. The difference in DAS28 increased over time [31] and was still present after 8 years of follow-up [32]. However, male patients had higher CRP at baseline [30] and there was no difference in baseline radiographic joint damage or progression of joint damage over time [31], although other studies have suggested that female gender may be an independent predictor of radiographic progression [33]. In the BeST study, a randomized controlled trial of 4 response-driven treatment strategies, female patients were significantly less likely to achieve drug-free remission [34]. PROs, including the HAQ, have also been noted to be worse in female patients compared to males in a large multinational database [35].

One possible explanation for such differences could be lower muscle mass in women. However, in our study, there were major differences in SF-36 scores compared to the general population for both sexes, with greater numerical differences for women.

The results of the present study are compatible with the concept that a higher pain perception in women is part of the explanation for these findings. This may reflect patterns that are not specific for RA, since chronic widespread pain is twice as frequent in women as in men in the general population [36], and female sex may be a predictor of future chronic widespread pain in individuals with regional pain [37]. On the other hand, the relative impact of RA on co-morbidity and mortality may be at least as great in women as in men. In a survey that included part of the population of the present study, treatment with TNF inhibitors was associated with a lower mortality in women, but not in men [28]. These differences are of major clinical relevance, and the underlying mechanisms should be further studied.

The major strengths of this study include the use of a community-based register of patients with RA diagnosis from a well-defined area, with structured assessment using repeated questionnaires during a period of 11 years.

Limitations include the sample size, which affects the precision of some of the outcome estimates, the lack of longitudinal data for analysis of individual cases, and the lack of available objective measures of disease activity due to the study design.

## Conclusions

In a well-defined population of patients with RA that received more aggressive treatment over time, we have demonstrated improvement in patient reported outcomes.

Despite similar treatment, male patients reported better outcomes and greater improvements over time, especially for pain and physical function. We suggest that patient reported outcomes should be reported separately in male and female patients with RA.

## Competing interests

The authors have no potential conflicts of interest regarding this paper.

## Authors’ contributions

KH performed the medical record review, participated in the design of the study and the statistical analysis, and drafted the manuscript. LJ and YL participated in the design of the study, developed the questionnaires and participated in the analysis and interpretation of data. JÅN participated in the design of the study and the statistical analysis. CT participated in the design of the study, assisted in the medical record review, participated in the statistical analysis and helped draft the manuscript. All authors read and approved the final manuscript.

## Pre-publication history

The pre-publication history for this paper can be accessed here:

http://www.biomedcentral.com/1471-2474/15/44/prepub

## References

[B1] Bergström U Book C Lindroth Y Marsal L Saxne T Jacobsson L Lower disease activity and disability in Swedish patients with rheumatoid arthritis in 1995 compared with 1978 Scand J Rheumatol 1999 28 160 165 10.1080/03009749950154239 10380838

[B2] Hekmat K Jacobsson L Nilsson JÅ Petersson IF Robertsson O Garellick G Turesson C Decrease in the incidence of total hip arthroplasties in patients with rheumatoid arthritis–results from a well-defined population in south Sweden Arthritis Res Ther 2011 13 R67 10.1186/ar3328 21510862 PMC3132062

[B3] Myasoedova E Crowson CS Turesson C Gabriel SE Matteson EL Incidence of extraarticular rheumatoid arthritis in Olmsted County, Minnesota, in 1995–2007 versus 1985–1994: a population-based study J Rheumatol 2011 38 6 983 989 10.3899/jrheum.101133 21459933 PMC3193155

[B4] Sokka T Kautiainen H Pincus T Toloza S da Rocha Castelar Pinheiro G Lazovskis J Hetland ML Peets T Immonen K Maillefert JF Drosos AA Alten R Pohl C Rojkovich B Bresnihan B Minnock P Cazzato M Bombardieri S Rexhepi S Rexhepi M Andersone D Stropuviene S Huisman M Sierakowski S Karateev D Skakic V Naranjo A Baecklund E Henrohn D Gogus F Disparities in rheumatoid arthritis disease activity according to gross domestic product in 25 countries in the QUEST-RA database Ann Rheum Dis 2009 68 11 1666 1672 10.1136/ard.2009.109983 19643759 PMC2756954

[B5] Pincus T Callahan LF Quantitative measures to assess, monitor and predict morbidity and mortality in rheumatoid arthritis Baillieres Clin Rheumatol 1992 6 161 191 10.1016/S0950-3579(05)80343-7 1563035

[B6] Yazici Y Bergman M Pincus T Time to score quantitative rheumatoid arthritis measures: 28-Joint Count, Disease Activity Score, Health Assessment Questionnaire (HAQ), Multidimensional HAQ (MDHAQ), and Routine Assessment of Patient Index Data (RAPID) scores J Rheumatol 2008 35 603 609 18322993

[B7] West E Jonsson SW Health-related quality of life in rheumatoid arthritis in Northern Sweden: a comparison between patients with early RA, patients with medium-term disease and controls, using SF-36 Clin Rheumatol 2005 24 117 122 10.1007/s10067-004-0976-6 15340864

[B8] Wallenius M Skomsvoll JF Koldingsnes W Rødevand E Mikkelsen K Kaufmann C Kvien TK Comparison of work disability and health-related quality of life between males and females with rheumatoid arthritis below the age of 45 years Scand J Rheumatol 2009 38 178 183 10.1080/03009740802400594 18991183

[B9] Arnett FC Edworthy SM Bloch DA McShane DJ Fries JF Cooper NS Healey LA Kaplan SR Liang MH Luthra HS The American Rheumatism Association 1987 revised criteria for the classification of rheumatoid arthritis Arthritis Rheum 1988 31 3 315 324 10.1002/art.1780310302 3358796

[B10] Jacobsson L Lindroth Y Marsal L Tejler L The Malmo model for private and public rheumatological outpatient care. Cooperation makes it possible to introduce disease modifying treatment quickly] Lakartidningen 2001 98 43 4710 4716 11715248

[B11] Söderlin MK Lindroth Y Turesson C Jacobsson LT A more active treatment has profound effects on the health status of rheumatoid arthritis (RA) patients: results from a population-based RA register in Malmö, Sweden, 1997–2005 Scand J Rheumatol 2010 39 206 211 10.3109/03009740903313621 20001765

[B12] Kvien TK Glennås A Knudsrød OG Smedstad LM Mowinckel P Førre O The prevalence and severity of rheumatoid arthritis in Oslo. Results from a county register and apopulation survey Scand J Rheumatol 1997 26 412 418 10.3109/03009749709065712 9433400

[B13] Ekdahl C Eberhardt K Andersson SI Svensson B Assessing disability in patients with rheumatoid arthritis. Use of a Swedish version of the Stanford Health Assessment Questionnaire Scand J Rheumatol 1988 17 263 271 10.3109/03009748809098795 3187457

[B14] Ware JE Jr CD S The MOS 36-item short-form health survey (SF-36). I. Conceptual framework and item selection. I. Conceptual framework and item selection Med Care 1992 30 473 483 10.1097/00005650-199206000-00002 1593914

[B15] Sullivan M Karlsson J Swedish manual and interpretation guide 1994 Gothenburg: Sahlgrenska University Hospital

[B16] Sullivan M Jan K John E Ware E The Swedish SF-36 Health Survey- I. Evaluation of data quality, scaling assumptions, reliability and construct validity across general populations in Sweden Soc Sci Med 1995 41 10 p1349 p1358 10p, 1 Diagram 10.1016/0277-9536(95)00125-Q 8560302

[B17] Pincus T Sokka T Kautiainen H Patients seen for standard rheumatoid arthritis care have significantly better articular, radiographic, laboratory, and functional status in 2000 than in 1985 Arthritis Rheum 2005 52 1009 1019 10.1002/art.20941 15818706

[B18] Krishnan E Fries JF Reduction in long-term functional disability in rheumatoid arthritis from 1977 to 1998: a longitudinal study of 3035 patients Am J Med 2003 115 371 376 10.1016/S0002-9343(03)00397-8 14553872

[B19] Uhlig T Heiberg T Mowinckel P Kvien TK Rheumatoid arthritis is milder in the new millennium: health status in patients with rheumatoid arthritis 1994–2004 Ann Rheum Dis 2008 67 1710 1715 10.1136/ard.2007.084673 18218667

[B20] Sokka T Kautiainen H Häkkinen K Hannonen P Radiographic progression is getting milder in patients with early rheumatoid arthritis. Results of 3 cohorts over 5 years J Rheumatol 2004 31 1073 1082 15170917

[B21] Grigor C Capell H Stirling A McMahon AD Lock P Vallance R Kincaid W Porter D Effect of a treatment strategy of tight control for rheumatoid arthritis (the TICORA study): a single-blind randomised controlled trial Lancet 2004 364 9430 263 269 10.1016/S0140-6736(04)16676-2 15262104

[B22] Verstappen SM Jacobs JW van der Veen MJ Heurkens AH Schenk Y ter Borg EJ Blaauw AA Bijlsma JW Utrecht rheumatoid arthritis cohort study group. Intensive treatment with Methotrexate in early rheumatoid arthritis: aiming for remission. Computer assisted management in early rheumatoid arthritis (CAMERA, an open-label strategy trial) Ann Rheum Dis 2007 66 11 1443 1449 Epub 2007 May 22 10.1136/ard.2007.071092 17519278 PMC2111604

[B23] Smolen JS Aletaha D Bijlsma JW Breedveld FC Boumpas D Burmester G Combe B Cutolo M de Wit M Dougados M Emery P Gibofsky A Gomez-Reino JJ Haraoui B Kalden J Keystone EC Kvien TK McInnes I Martin-Mola E Montecucco C Schoels M Van der Heijde D T2T Expert Committee Treating rheumatoid arthritis to target: recommendations of an international task force Ann Rheum Dis 2010 69 631 637 10.1136/ard.2009.123919 20215140 PMC3015099

[B24] Smolen JS Landewé R Breedveld FC Buch M Burmester G Dougados M Emery P Gaujoux-Viala C Gossec L Nam J Ramiro S Winthrop K de Wit M Aletaha D Betteridge N Bijlsma JW Boers M Buttgereit F Combe B Cutolo M Damjanov N Hazes JM Kouloumas M Kvien TK Mariette X Pavelka K van Riel PL Rubbert-Roth A Scholte-Voshaar M Scott DL EULAR recommendations for the management of rheumatoid arthritis with synthetic and biological disease-modifying antirheumatic drugs Ann Rheum Dis 2010 69 964 975 10.1136/ard.2009.126532 20444750 PMC2935329

[B25] Turesson C Baecklund E Forsbladd’Elia H Guidelines for the pharmaceutical management of rheumatoid arthritis Swed Soc Rheumatol 2011 http://www.svenskreumatologi.se

[B26] Sokka T Haugeberg G Asikainen J Widding Hansen IJ Kokko A Rannio T Soldal DM Hannonen P Similar clinical outcomes in rheumatoid arthritis with more versus less expensive treatment strategies. Observational data from two rheumatology clinics Clin Exp Rheumatol 2013 31 3 409 414 23415074

[B27] Wolfe F Mitchell DM Sibley JT Fries JF Bloch DA Williams CA Spitz PW Haga M Kleinheksel SM Cathey MA The mortality of rheumatoid arthritis Arthritis Rheum 1994 37 481 94 10.1002/art.1780370408 8147925

[B28] Jacobsson LT Turesson C Nilsson JA Petersson IF Lindqvist E Saxne T Geborek P Treatment with TNF blockers and mortality risk in patients with rheumatoid arthritis Ann Rheum Dis 2007 66 670 5 10.1136/ard.2006.062497 17158824 PMC1954627

[B29] Weyand CM Schmit D Wagner U Goronzy JJ The influence of sex on the phenotype of rheumatoid arthritis Arthriris Rheum 1998 41 817 822 10.1002/1529-0131(199805)41:5<817::AID-ART7>3.0.CO;2-S 9588732

[B30] Forslind K Hafström I Ahlmén M Svensson B BARFOT Study Group Sex: a major predictor of remission in early rheumatoid arthritis? Ann Rheum Dis 2007 66 46 52 17158139 10.1136/ard.2006.056937 PMC1798403

[B31] Ahlmén M Svensson B Albertsson K Forslind K Hafström I BARFOT Study Group Influence of gender on assessments of disease activity and function in early rheumatoid arthritis in relation to radiographic joint damage Ann Rheum Dis 2010 69 230 3 10.1136/ard.2008.102244 19158113

[B32] Hafström I Vaentina B Albertsson K Forslind K Svensson B Joint destruction in early rheumatoid arthritis over 8 years is similar in woman and men despite apparently higher disease activity and poorer sunction in women Ann Rheum Dis 2011 70 709 710 10.1136/ard.2010.135343 20699238

[B33] Syversen SW Gaarder PI Goll GL Ødegård S Haavardsholm EA Mowinckel P van der Heijde D Landewé R Kvien TK High anti-cyclic citrullinated peptide levels and an algorithm of four variables predict radiographic progression in patients with rheumatoid arthritis: results from a 10-year longitudinal study Ann Rheum Dis 2008 67 212 7 10.1136/ard.2006.068247 17526555

[B34] der Kooij SM Goekoop-Ruiterman YP de Vries-Bouwstra JK Güler-Yüksel M Zwinderman AH Kerstens PJ van der Lubbe PA de Beus WM Grillet BA Ronday HK Huizinga TW Breedveld FC Dijkmans BA Allaart CF Drug-free remission, functioning and radiographic damage after 4 years of responsedriven treatment in patients with recent-onset rheumatoid arthritis Ann Rheum Dis 2009 68 914 21 10.1136/ard.2008.092254 18662933

[B35] Sokka T Toloza S Cutolo M Kautiainen H Makinen H Gogus F Skakic V Badsha H Peets T Baranauskaite A Géher P Ujfalussy I Skopouli FN Mavrommati M Alten R Pohl C Sibilia J Stancati A Salaffi F Romanowski W Zarowny-Wierzbinska D Henrohn D Bresnihan B Minnock P Knudsen LS Jacobs JW Calvo-Alen J Lazovskis J Pinheiro Gda R Karateev D Women, men, and rheumatoid arthritis: analyses of disease activity, disease characteristics, and treatments in the QUEST-RA Study Arthritis Res Ther 2009 11 R7 doi:10.1186/ar2591 10.1186/ar2591 PMC2688237 19144159

[B36] Bergman S Herrström P Högström K Petersson IF Svensson B Jacobsson LT Chronic musculoskeletal pain, prevalence rates, and sociodemographic associations in a Swedish population study J Rheumatol 2001 28 1369 77 11409133

[B37] Bergman S Herrström P Jacobsson LTH Petersson IF Chronic widespread pain: a three-year followup of pain distribution and risk factors J Rheumatol 2002 29 818 25 11950027

